# Effects of Bunch Rot (*Botrytis cinerea*) and Powdery Mildew (*Erysiphe necator*) Fungal Diseases on Wine Aroma

**DOI:** 10.3389/fchem.2017.00020

**Published:** 2017-03-28

**Authors:** Angela Lopez Pinar, Doris Rauhut, Ernst Ruehl, Andrea Buettner

**Affiliations:** ^1^Department of Chemistry and Pharmacy, Emil Fischer Center, Friedrich-Alexander-Universität Erlangen-NürnbergErlangen, Germany; ^2^Department of Microbiology and Biochemistry, Hochschule Geisenheim UniversityGeisenheim, Germany; ^3^Department of Grapevine Breeding, Hochschule Geisenheim UniversityGeisenheim, Germany; ^4^Department Sensory Analytics, Fraunhofer Institute for Process Engineering and Packaging IVVFreising, Germany

**Keywords:** gas chromatography-olfactometry GC-O, aroma extract dilution analysis AEDA, sensory analysis, lactone, 2-phenylethanol, isoamyl alcohol

## Abstract

This study aimed to characterize the effects of bunch rot and powdery mildew on the primary quality parameter of wine, the aroma. The influence of these fungal diseases was studied by comparative Aroma Extract Dilution Analyses (AEDA) and sensory tests. The effect of bunch rot was investigated on three grape varieties, namely White Riesling, Red Riesling and Gewürztraminer and that of powdery mildew on the hybrid Gm 8622-3; thereby, samples were selected that showed pronounced cases of infection to elaborate potential currently unknown effects. Both infections revealed aromatic differences induced by these fungi. The sensory changes were not associated with one specific compound only, but were due to quantitative variations of diverse substances. Bunch rot predominantly induced an increase in the intensities of *peach-like/fruity, floral* and *liquor-like/toasty* aroma notes. These effects were found to be related to variations in aroma substance composition as monitored via AEDA, mainly an increase in the FD factors of lactones and a general moderate increase of esters and alcohols. On the other hand, powdery mildew decreased the *vanilla-like* character of the wine while the remaining sensory attributes were rather unaffected. Correspondingly, FD factors of the main aroma constituents were either the same or only slightly modified by this disease. Moreover, bunch rot influenced the aroma profiles of the three varieties studied to a different degree. In hedonic evaluation, bunch rot-affected samples were rated as being more pleasant in comparison to their healthy controls in all three varieties while the powdery mildew-affected sample was rated as being less pleasant than its healthy control.

## Introduction

Fungal diseases are an economic threat for viticulture. An increase in fungal infections has been related to climate change as this phenomenon may provide more favorable conditions for fungi to grow (Chakraborty et al., 2000). Thereby, warm-moderate temperatures and high relative humidity favor the development of *Botrytis cinerea* and *Erysiphe necator* (Schnathorst, 1965; Thomas et al., 1988; Caffarra et al., 2012). Moreover, the usage of fungicides is increasingly restricted favoring the spread of fungal diseases (EUR-lex^1^; Gullino and Kuijpers, 1994).

This study focused on *Botrytis cinerea* and *Erysiphe necator*, since they are amongst the most relevant fungi in viticulture and have a world-wide impact on the wine industrial production (Dean et al., 2012; Steel et al., 2013). They are responsible for bunch rot and powdery mildew diseases, respectively. Both fungi can affect grape berries, resulting in yield losses and reduced fruit quality depending on the degree of infection (Stummer et al., 2003, 2005; Calonnec et al., 2004; Steel et al., 2013).

Effects of bunch rot on wine quality are: (I) an altered carbohydrate metabolism leading to an increase in the amount of high molecular weight polysaccharides causing problems during processing; (II) an enhanced production of the oxidative enzyme laccase that has been shown to be related to a browning effect in wine color; (III) the generation of off-odors, and (IV) a reduction of the concentration of phenolic compounds, organic acids and varietal aromas (Ribéreau Gayon et al., 1980; Slomczynski et al., 1995; Ky et al., 2012; Steel et al., 2013).

While the effects of *Botrytis* bunch rot are well defined and thoroughly investigated (Steel et al., 2013), those of powdery mildew are still less understood. Nonetheless, it has been described that this disease causes a decrease in grape soluble solids, negatively impacts wine color, and causes higher acidity (Pool et al., 1984; Gadoury et al., 2001; Stummer et al., 2003, 2005; Calonnec et al., 2004). In addition, it has been reported to be responsible for mushroom-like off-odors (Darriet et al., 2002; Stummer et al., 2005).

This study centered on the analysis of the changes caused by these fungi on wine aroma. For this aim, Aroma Extract Dilution Analysis (AEDA) (Grosch, 2001) was performed to screen for those substances with the highest potential to impact wine aroma amongst a multitude of volatile compounds present in wine. Besides, this approach allows establishing a preliminary ranking of the aroma active compounds according to their relative olfactory intensity. First and foremost, however, it enables a direct comparison of the relative intensities of specific odorants between samples when being conducted as comparative AEDA. This technique has been successfully applied in previous studies to monitor the main differences in odorant composition in, for example, fresh and processed orange juice (Buettner and Schieberle, 2001a), and in stored human milk in comparison to freshly sampled milk (Spitzer and Buettner, 2010). In the present study, we used this technique to compare the odorant composition and potential chemical changes between samples produced from healthy and fungi-infected grapes using four extreme cases of fungal infection as models to elaborate major changes that can be used later for enologically relevant samples.

This article is based on our previous study where effects of these two fungi on grape must aroma were investigated (Lopez Pinar et al., 2016). The present study builds on the wine obtained from these aforementioned must samples and, accordingly, intends to complement the knowledge of fungal aroma effects, starting from the harvested raw material to the final product. Specifically, the aim was to characterize in a model situation, the effects of bunch rot infection on wine produced from White Riesling, Red Riesling and Gewürztraminer grape varieties, and the effects of powdery mildew on wine produced from the unsprayed hybrid Gm 8622-3.

## Materials and methods

### Chemicals

Dichloromethane and anhydrous sodium sulfate were purchased from VWR (Darmstadt Germany). The reference substances were obtained from the following suppliers: ethyl isobutanoate, ethyl 2-methylbutanoate, 3-methyl-1-butanol (isoamyl alcohol), ethyl hexanoate, (*Z*)-3-hexen-1-ol, (*E*)-2-octenal, acetic acid, 3-(methylthio)-propanal (methional), 3,7-dimethyl-1,6-octadien-3-ol (linalool), 3-methylbutanoicacid, 1,4-diethylbutanedionate (diethyl succinate), 3-(methylthio)-1-propanol (methionol), (*E*)-3,7-dimethyl-2,6-octadien-1-ol-acetate (geranylacetate), phenylacetate, hexanoic acid, benzyl alcohol, 2-phenylethanol, γ-nonalactone, 4-ethyl-2-methoxyphenol(4-ethylguaiacol), octanoic acid, 5(or 2)-ethyl-4-hydroxy-2(or 5)-methyl-3(2*H*)-furanone (homofuraneol), 3-methylphenol (m-cresol), γ-decalactone, 3-hydroxy-4,5-dimethyl-2(5*H*)-furanone (sotolone), 4-ethylphenol, 4-ethenyl-2-methoxyphenol (4-vinylguaiacol), decanoic acid, (4*S*,4a*S*,8a*R*)-4,8a-dimethyl-1,2,3,4,5,6,7,8-octahydronaphtalen-4a-ol [(±)-geosmin], γ-undecalactone and phenylacetic acid were purchased from Sigma-Aldrich (Steinheim, Germany). Ethyl butanoate, 2,3-butanedione (diacetyl), ethyl 3-methylbutanoate (ethyl isovalerate), 2-methyl-1-propanol (isobutanol), 3-methylbutyl acetate, 1-hexanol, butanoic acid, 2-phenethyl acetate and 4-hydroxy-2,5-dimethyl-3(2*H*)-furanone (furaneol) were obtained from Fluka (Steinheim, Germany). The reference compounds 4-mercapto-4-methyl-2-pentanone and 4-hydroxy-3-methoxybenzaldehyde (vanillin) were supplied by ABCR (Karlsruhe, Germany). Ethyl octanoate was obtained from Alfa Aesar (Karlsruhe, Germany). Propanoic acid was from Riedel-de Haen (Seelze, Germany). Undecanoic acid and 2-methylpropanoic acid (isobutanoic acid) were purchased from SAFC (Steinheim, Germany). Finally, (*Z*)-6-dodeceno-γ-lactone was purchased from Aromalab (Freising, Germany).

### Wine preparation

Grapes were harvested in Geisenheim (Germany) in October 2014. To study the influence of *Botrytis* bunch rot infection, White Riesling, Red Riesling and Gewürztraminer grapes were used. The effects of powdery mildew were investigated on the hybrid Gm 8622-3. In each case, healthy berry samples and samples affected by the fungus at a very high degree were compared. Moreover, in the case of White Riesling, an additional intermediate state of bunch rot infection was included. In each case, the healthy and the infected sample were collected separately, but both samples were harvested the same day in the same vineyard and were later processed identically. The degree of fungal infection on the grape berries was visually assessed as described in our previous study (Lopez Pinar et al. (2016), providing photographs with representative examples of grapes affected by bunch rot and powdery mildew).

For must preparation, grapes were crushed and then pressed in a pneumatic press and left to settle with 50 mg/L SO_2_ for approximately 24 h at 10°C, followed by filtering through filter paper. The grape juices were subsequently fermented at 17°C for 2–3 weeks long in 20 L glass balloons by inoculation of the reactivated pure yeast culture *Saccharomyces cerevisiae* (var. *bayanus*) Lalvin EC 1118 (Lallemand, Montreal, Canada). After fermentation, wines were racked and the concentration of SO_2_ was adjusted to approximately 50 mg/L free SO_2_ by addition of an aqueous solution of SO_2_ (5%) purchased from Baldinger (Fällander, Switzerland). Finally, samples were sterile filtered and bottled in brown 0.75 L glass bottles which were closed with screw caps.

### Extraction of the volatile compounds

In order to extract the volatile compounds, 100 mL of wine were mixed with 50 mL dichloromethane and stirred for 1 h at room temperature. Then, the mixture was submitted to solvent assisted flavor evaporation (SAFE) (Engel et al., 1999) for the isolation of the volatiles; the obtained dichloromethane phase was separated and dried over anhydrous sodium sulfate. Finally, the extract was concentrated at 51°C by means of Vigreux distillation and micro-distillation to a final volume of 200 μL.

### Gas chromatography-olfactometry analysis (GC-O)

Analyses were performed on a GC type Trace Ultra (Thermo Finnigan, Dreieich, Germany) using the capillaries DB-FFAP and DB-5 (30 m, 0.32 mm, film thickness 0.25 μm; J&W Scientific, Fisons Instruments, Mainz-Kastel, Germany). The samples were applied by the cold-on-column technique, 2 μL of the sample were manually injected at 40°C directly into a pre-column, which consisted of an uncoated, deactivated fused silica capillary (2–3 m, 0.32 mm). In case that a DB-FFAP capillary was used, the initial temperature was held for 2 min, then the temperature of the GC oven was raised with 8°C/min to 240°C and held for 5 min. In case of usage of a DB-5 capillary, the temperature program was as follows: the initial 40°C were held for 2 min, then the temperature was raised with 4°C/min to 90°C, then with 8°C/min to 220°C, and finally with 20°C/min to 300°C, and held for 2 min. The flow rate of the helium carrier gas was 2 mL/min. At the end of the capillary, the effluent was split between a sniffing port and a flame ionization detector (FID) using two deactivated but uncoated fused silica capillaries (70 cm, 0.32 mm). The FID and the sniffing port were held at 250°C.

### Aroma extract dilution analysis (AEDA)

The aroma active compounds were ranked according to their intensities by means of comparative AEDA (Buettner and Schieberle, 2001a; Grosch, 2001). The initial extract was stepwise diluted in the ratio 1+1 (v/v) with dichloromethane, and each dilution step was analyzed by GC-O until no odor was detected. The flavor dilution (FD) factor is defined as the last dilution step at which the compound could be detected. The complete AEDA was performed per duplicate.

### High resolution gas chromatography–mass spectrometry (GC–MS)

Mass spectra were obtained on two different instruments: The measurements on the DB-5 column were performed on a 5973 MSD quadrupole system (Hewlett-Packard, Palo Alto, CA, United States) fitted to a 6890 GC (Agilent Technologies, Waldbronn, Germany), while for the measurements on the DB-FFAP capillary column, a 5975C MSD quadrupole system combined with a 7890A GC system (Agilent Technologies) was used. Both were equipped with Gerstel CIS injection system models 3 and 4, for the first and the second system, respectively. In addition, a Gerstel MPS 2 auto sampler (Gerstel, Duisburg, Germany) was used. The dimensions of the analytical capillaries DB-FFAP and DB-5 were 30 m, 0.25 mm, film thickness 0.25 μm (J&W Scientific). An uncoated, deactivated fused silica capillary was used as pre-column (2–3 m, 0.53 mm). The carrier gas was helium and the flow rate was 1.0 and 1.2 mL/min for measurements on DB-FFAP and DB-5, respectively. EI-mass spectra were generated in full scan mode (m/z range 40–400) at 70 eV ionization energy. The scan rate was 3.94 scans/s. Injection volume was 1 μL in each case. The temperature program used for DB-FFAP measurements was as follows: the initial 40°C were held for 2 min, then the temperature was raised with 8°C/min to 240°C and held for 5 min. In case of DB-5, the initial 40°C were held for 2 min, then the temperature was raised with 8°C/min to 240°C, and finally the temperature was increased with 20°C/min until 300°C was reached, and the final temperature was held for 5 min.

### Two-dimensional high resolution gas chromatography–mass spectrometry/olfactometry (HRGC–GC–MS/O)

For mass spectrometric identification of trace constituents, a two-dimensional gas chromatographic system was applied. It consisted of two Varian 450-GCs (Varian, Darmstadt, Germany) combined with a Saturn 2,200 MS (Varian). The first GC was equipped with a Gerstel multi-column switching system MCS 2 and connected to the second GC by a Gerstel cryo-trap system CTS 1 (Gerstel). The system was equipped with a Gerstel CIS 3 injection system and Gerstel MPS 2 auto sampler (Gerstel). The capillaries used were a DB-FFAP capillary column (J&W Scientific, with the dimensions described in GC-O section in the first oven and in the second oven a Rxi®-5HT (30 m, 0.25 mm, film thickness 0.25 μm; Restek, Homburg, Germany). The temperature programs were as follows: initial 40°C were held for 2 min, then the temperature was increased with 8°C/min to a final temperature of 240°C (for DB-FFAP measurements) or 250°C (for Rxi®-5HT measurements), that was held for 5 min. At the end of the capillary, the effluent was split between a sniffing port and a FID, or a MS, in the first and second oven, respectively, using two deactivated but uncoated fused silica capillaries (100 cm, 0.20 mm). Application of the samples to the GC system was performed at 40°C using the cold-on-column technique. The flow rate of the helium carrier gas was 2.0 mL/min. The FID and the sniffing port were held at 240 and 260°C, respectively. Mass spectra were generated at 70 eV ionization energy in electron ionization (EI) mode (m/z range 30–300), or in the positive chemical ionization (CI) mode with methanol as the reactant gas (m/z range 35–300).

### Sensory evaluation

The sensory panel consisted of 10 participants (1 male and 9 females, 25–35 years old) (Buettner and Schieberle, 2001b; Spitzer et al., 2013). Panelists were previously trained for at least 6 months in weekly sessions in recognizing about 90 selected odorants and in naming these according to an in-house developed flavor language. Preliminary sensory sessions were held in order to establish the aroma attributes that most accurately described the samples. Based on these pre-evaluations, seven aroma attributes were selected as most commonly named descriptors and were rated on a scale from 0 (not perceived) to 3 (very intensively perceived); thereby, intermediate steps of 0.5 were allowed. During the sensory tests, participants had aqueous reference solutions at their disposal that related to the provided attributes that should be rated: 2-phenylethanol for *floral*, (*Z*)-6-dodeceno-γ-lactone for *peach-like/fruity*, butanoic acid for *cheesy*, isoamyl alcohol for *liquor-like/toasty*, acetic acid for *vinegar-like*, geosmin for *musty*, and vanillin for *vanilla-like* smells. Pleasantness of the samples was also evaluated on a scale from 0 (extremely disagreeable) to 3 (very pleasant); intermediate steps of 0.5 were allowed.

The standard deviation of the hedonic results was calculated via Excel. The statistical significance of the effects of the two fungal diseases on the sensory evaluation was analyzed based on a comparison of the mean rating values from affected and unaffected samples using Student's *t*-test (significance level of *p* < 0.05).

## Results

### AEDA

A total of 51 odor active compounds were detected by GC-O in the nine wine samples analyzed within this study (Table 1). Substances predominantly exhibited *fruity* smells whereas the *rose-like* smelling substance 2-phenylethanol and the *liquor/chocolate-like* smelling isoamyl alcohol were, in case of all samples, found with the highest FD factors in the range from 1,024 to 8,192.

**Table 1:** **Results of the Aroma Extract Dilution Analysis (AEDA)**.

**Substance**	**Odor attributes^a^**			**Identification parameter^c^**	**FD^d^**								
					**Bunch rot**							**Powdery mildew**	
		**RI^b^ on**			**White Riesling**			**Red Riesling**		**Gewürz traminer**		**Gm 8622-3**	
		**DB-FFAP**	**DB5**		**HW**	**IW**	**BW**	**HW**	**BW**	**HW**	**BW**	**HW**	**PW**
Ethyl isobutanoate	Berry	≤1,000	717	O, RI, MS^2^, S	512	256	512	64	2,048	128	128	512	64
2,3-Butanedione (Diacetyl)	Buttery	1,021	622	O, RI, MS^2*^, S	32	64	256	16	256	64	64	64	128
Ethyl butanoate	Berry	1,050	767	O, RI, MS^1^, S	64	128	512	256	1,024	512	128	64	256
Ethyl 2-methylbutanoate	Berry, banana	1,064	852	O, RI, MS^2^, S	256	512	512	512	1,024	128	128	512	64
Ethyl 3-methylbutanoate (Ethyl isovalerate)	Berry, apple	1,088	822	O, RI, MS^1^, S	32	16	4	4	64	8	n.d.^f^	n.d.^f^	16
2-Methyl-1-propanol (Isobutanol)	Liquor, chocolate	1,100	667	O, RI, MS^1^, S	128	256	128	128	1,024	64	128	64	128
3-Methylbutyl acetate	Banana	1,125	885	O, RI, MS^1^, S	64	64	128	64	128	256	128	1,024	128
3-Methyl-1-butanol (Isoamyl alcohol)	Liquor, chocolate	1,212	744	O, RI, MS^1^, S	2,048	2,048	8,192	2,048	8,192	1,024	2,048	4,096	2,048
Ethyl hexanoate	Berry, mint	1,241	1,000	O, RI, MS^1^, S	512	256	512	128	128	128	256	1,024	512
1-Hexanol	Soapy, floral, cherry	1,353	804	O, RI, MS^1^, S	2	16	8	8	4	32	64	32	16
(*Z*)-3-Hexen-1-ol	Green, fatty, mint	1,394	n.d.^e^	O, RI, MS^1^, S	8	n.d.^f^	n.d.^f^	n.d.^f^	n.d.^f^	n.d.^f^	n.d.^f^	n.d.^f^	4
4-Mercapto-4-methyl-2-pentanone	Cat urine	1,400	947	O, RI, S	32	32	64	2	64	8	4	2	n.d.^f^
(*E*)-2-Octenal	Soapy	1,413	n.d.^e^	O, RI, S	16	2	8	2	4	16	64	32	8
unknown	Cheesy, beer	1,427	903		64	128	16	64	128	16	64	32	32
unknown	Cheesy	1,433	1,097		256	128	256	128	8	n.d.^f^	128	16	16
Ethyl octanoate	Dust, soapy	1,447	1,205	O, RI, MS^1^, S	8	8	2	16	2	32	32	16	8
Acetic acid	Vinegar	1,460	644	O, RI, MS^1^, S	512	128	1,024	128	1,024	128	512	256	256
3-(Methylthio)-propanal (Methional)	Cooked potato	1,480	909	O, RI, S	128	128	256	256	256	64	512	128	128
Propanoic acid	Cheesy	1,507	n.d.^e^	O, RI, MS^1^, S	256	128	256	n.d.^f^	16	n.d.^f^	32	512	512
3,7-Dimethyl-1,6-octadien-3-ol (Linalool)	Acid, soapy	1,533	n.d.^e^	O, RI, MS^1^, S	n.d.^f^	8	n.d.^f^	32	8	8	8	32	8
2-Methylpropanoic acid (Isobutanoic acid)	Rancid, cheesy	1,560	870	O, RI, MS^1^, S	16	2	1	64	4	64	n.d.^f^	32	32
unknown	Caramel, coffee	1,607	1,209		4	4	2	n.d.^f^	8	1	8	2	8
Butanoic acid	Cheesy	1,629	n.d.^e^	O, RI, MS^1^, S	128	32	64	32	64	32	32	128	128
3-Methylbutanoic acid	Cheesy, rancid	1,664	859	O, RI, MS^1^, S	1,024	256	128	256	512	256	64	512	128
unknown	Onion	1,693	953		128	16	16	256	8	16	16	64	256
1,4-Diethyl butanedioate (Diethyl succinate)	Plastic, dusty, soapy	1,707	n.d.^e^	O, RI, MS^1^, S	16	8	2	n.d.^f^	8	n.d.^f^	2	8	n.d.^f^
3-(Methylthio)-1-propanol (Methionol)	Cooked potato, onion	1,715	982	O, RI, MS^1^, S	128	128	512	64	1,024	64	64	128	128
(*E*)-3,7-Dimethyl-2,6-octadienyl acetate (Geranyl acetate)	Fatty, green, soapy	1,777	n.d.^e^	O, RI, S	4	4	1	2	8	8	16	n.d.^f^	4
Phenylacetate	Floral	1,792	n.d.^e^	O, RI, MS^1^, S	4	1	8	2	4	4	32	n.d.^f^	4
2-Phenethyl acetate	Honey, grape	1,823	n.d.^e^	O, RI, MS^1^, S	16	32	16	8	512	32	32	32	64
Hexanoic acid	Cheesy, rancid	1,838	1,091	O, RI, MS^1^, S	128	1,024	1,024	512	2,048	512	1,024	512	512
Benzyl alcohol	Grape	1,900	n.d.^e^	O, RI, MS^1^, S	512	512	2,048	64	1,024	32	256	1,024	512
2-Phenylethanol	Rose	1,907	1,114	O, RI, MS^1^, S	2,048	2,048	8,192	2,048	8,192	1,024	4,096	4,096	2,048
γ-Nonalactone	Woody, grape, coconut	2,000	1,350	O, RI, MS^2^, S	16	2	16	16	64	16	64	1	4
4-Ethyl-2-methoxyphenol (4-Ethylguaiacol)	Spicy, smokey	2,008	n.d.^e^	O, RI, S	128	128	64	32	128	16	32	64	64
4-Hydroxy-2,5-dimethyl-3(*2H*)-furanone (Furaneol)	Caramel	2,033	1,074	O, RI, S	256	256	128	256	1,024	128	512	512	128
Octanoic acid	Plastic, dusty	2,067	1,300	O, RI, MS^1^, S	16	32	8	64	8	32	32	32	16
5 (or 2)-Ethyl-4-hydroxy-2 (or 5)-methyl-3(*2H*)-furanone (Homofuraneol)	Caramel	2,083	1,154	O, RI, S	512	1,024	2,048	256	1,024	512	2,048	2,048	1,024
3-Methylphenol (m-Cresol)	Leather	2,109	n.d.^e^	O, RI, S	2	2	2	n.d.^f^	4	8	4	4	32
γ-Decalactone	Grape, peach	2,145	1,476	O, RI, MS^2*^, S	256	1,024	8,192	512	512	256	512	1,024	1,024
3-Hydroxy-4,5-dimethyl-2(*5H*)-furanone (Sotolone)	Smokey, curry	2,173	1,125	O, RI, S	16	8	512	16	2,048	16	64	64	256
4-Ethylphenol	Gummy, ink	2,200	1,179	O, RI, S	n.d.^f^	8	8	64	32	8	32	8	n.d.^f^
4-Ethenyl-2-methoxyphenol (4-Vinylguaiacol)	Smokey	2,218	1,311	O, RI, MS^2^, S	512	1,024	128	256	128	256	512	512	64
unknown	Woody, coconut, minty	2,245	1,471		256	256	2	64	1,024	128	128	8	16
Decanoic acid	Plastic, dusty	2,264	1,389	O, RI, MS^1^, S	64	16	32	32	128	16	64	128	16
γ-Undecalactone	Grape, peach	2,282	1,593	O, RI, S	128	256	256	64	2,048	8	32	16	32
Undecanoic acid	Green, fatty, leafy	2,350	n.d.^e^	O, RI, S	8	8	8	8	4	4	1	1	1
(*Z*)-6-Dodeceno-γ-lactone	Peach	2,430	1,664	O, RI, S	64	128	128	64	1,024	32	64	32	8
Phenylacetic acid	Honey	2,580	1,277	O, RI, MS^1^, S	512	1,024	1,024	512	2,048	512	512	256	512
4-Hydroxy 3-methoxy benzaldehyde (Vanillin)	Vanilla	2,590	1,412	O, RI, S	128	256	2,048	128	128	64	1,024	256	32
Unknown	Smokey	2,610	1,533		4	32	4	1	4	4	n.d.^f^	n.d.^f^	n.d.^f^

*Comparison of healthy samples (HW) and samples affected by bunch rot (BW) and powdery mildew (PW), respectively. For White Riesling, an additional intermediate state of bunch rot affection (IW) was investigated*.

a *Odor quality as perceived at the sniffing port*.

b *Retention indices according to Kovats (1958)*.

c Compounds were identified via the following criteria; O, Odor quality; RI, retention indices on the named capillary columns; MS^1^, EI-mass spectrum measured on one-dimensional GC-MS; MS^2^, EI-mass spectrum measured on two-dimensional GC-MS; MS^2*^, CI-mass spectrum measured on two-dimensional GC-MS; S, comparison with reference.

d *Flavor dilution (FD) factor on the capillary column FFAP*.

e *n.d., not determined due to: unsatisfactory chromatographic separation on this analytical column or inconclusive assignment of the smell to a specific retention factor due to co-elution with other odor-active substances*.

f *n.d., not detectable*.

Of all detected odorants, 31 odorants reflecting the substances with the highest FD factors were identified based on the following criteria: linear retention indices on two capillary columns with different polarity (Kovats, 1958), mass spectra and odor qualities in relation with the relative reference substances. For 25 of these odorants, mass spectrometric identification was achieved using one-dimensional GC-MS; these compounds were eight esters (ethyl butanoate, ethyl isovalerate, 3-methylbutyl acetate, ethyl hexanoate, ethyl octanoate, diethyl succinate, phenyl acetate and 2-phenethyl acetate), nine acidic compounds (acetic, propanoic, isobutanoic, butanoic, 3-methylbutanoic, hexanoic, octanoic, decanoic, and phenylacetic acid) and eight alcohols (isobutanol, isoamyl alcohol, 1-hexanol, (*Z*)-3-hexen-1-ol, linalool, methionol, benzyl alcohol, and 2-phenylethanol). For another six compounds, however, it was not possible to obtain mass spectrometric data with sufficient resolution in one-dimensional GC-MS, due to their low concentrations or coelution problems, respectively. Nevertheless, clear mass spectrometric data was achieved when using HRGC–GC–MS/O allowing successful identification of γ-decalactone, γ-nonalactone, 2,3-butanedione, ethyl isobutanoate, ethyl 2-methylbutanoate and 4-vinylguaiacol.

In case of γ-undecalactone, vanillin and methional, the match factor (reverse mode) of the mass spectra obtained in the two-dimensional GC-MS analyses with those of the corresponding reference substance was 720 only for γ-undecalactone, 766 for vanillin and 673 for methional, respectively, due to their low concentration levels. The highest value of this parameter is 1,000, corresponding to perfect match. Accordingly, these compounds are reported here as tentatively identified based on the remaining criteria in comparison to the corresponding reference substances.

In case of another 11 odorants, it was not possible to obtain a mass spectrum with sufficient resolution, neither in one-, nor in two-dimensional GC-MS analysis, due to their very low concentrations in the samples. As a result, these compounds are also reported here as tentatively identified based on their characteristic odor impressions and their retention indices: 4-mercapto-4-methyl-2-pentanone, (*E*)-2-octenal, geranyl acetate, 4-ethylguaiacol, furaneol, homofuraneol, m-cresol, sotolone, 4-ethylphenol, undecanoic acid, (*Z*)-6-dodeceno-γ-lactone.

Thereby, the most relevant tentatively identified substances in terms of FD factors exhibited very characteristic smells: furaneol and homofureaneol both smell like *caramel*, sotolone like *curry, savory-like*, methional like *cooked potato*, γ-undecalactone and (*Z*)-6-dodeceno-γ-lactone like *peach* and vanillin like *vanilla*, respectively. Accordingly, their characteristic odor is a strong identification criterion, so that this parameter together with the respective retention index data provides a solid foundation for their identification.

Lastly, the identification of the remaining six compounds was not possible based on these criteria as they did not match any known substance of our comprehensive in-house odorant database, and as insufficient mass spectrometric data was obtained in the course of our determinations. Nevertheless, these unidentified substances were mostly of no major relevance in terms of differences between healthy and fungi-infected samples. Exceptions will be specified later on.

In the next step of the analyses, the FD factors of the potent odorants in the healthy wine samples (HW) were compared to those obtained for the same substances in the samples made from grapes affected by *Botrytis* bunch rot (BW) or powdery mildew (PW) to a very high degree, in order to more closely elaborate the effects of the respective fungal infection on the final wine aroma. For the variety White Riesling, an additional intermediate state of bunch rot infection (IW) was investigated. These wine samples relate to the corresponding must samples as previously investigated in Lopez Pinar et al. (2016).

In the following, the diverse effects of these types of infection on the odorant composition of the wines will be discussed in relation to specific substances classes. Overall, only major changes in FD factors will be discussed in detail, as slight variations in one or two FD steps are within the natural variation of the olfactometric detection.

### Effects of bunch rot infection

With regards to the group of acids, a great decrease in FD factors of isobutanoic acid due to bunch rot was observed with 4-,4-, and 7-fold lower dilution steps in White Riesling, Red Riesling and Gewürztraminer, respectively (Table 1). A different effect was observed for propanoic acid: its FD factor was remarkably increased in BW by five dilution steps in Red Riesling and by six steps in Gewürztraminer, while the intensity level of this substance was not affected in White Riesling. For the remaining acidic substances, variable effects were observed with slight variations by one or two FD steps in each case only.

The FD factors of the esters were in most cases moderately increased due to bunch rot infection. However, a major increase of ethyl isobutanoate was observed in Red Riesling with its FD factor being increased by five dilution steps in BW. On the other hand, this ester remained unaffected in the other two varieties.

Regarding the alcoholic substances, a general increase in their FD-factors was observed comprising, however, mostly slight variations of up to two dilution steps. Nevertheless, there were two relevant changes that need to be pointed out: it was observed that bunch rot induced a major increase in the FD factors of four dilution steps in the Red Riesling variety for methionol and benzyl alcohol. However, no major changes were found for these compounds in the other varieties.

In addition, major intensity changes were observed for the lactones. Overall, bunch rot led to an increase in their FD factors, but the extent of this effect was different among the varieties studied. A major increase of γ-decalactone comprising five dilution steps was detected in White Riesling, and γ-undecalactone and (Z)-6-dodeceno-γ-lactone were both increased by four dilution steps in Red Riesling. The intensities of the same substances were also moderately elevated in the other varieties, as was the FD factor of γ-nonalactone in BW. Moreover, bunch rot caused a relevant increase in the FD factors of sotolone in case of all investigated wine varieties: on average, they were four dilution steps higher in BW than in their corresponding healthy samples.

As representative of the group of potent thio-odorants, the cat urine-like smelling 4-mercapto-4-methyl-2-pentanone was greatly increased in Red Riesling: its FD factor was five dilution steps higher in BW. In the other two varieties, however, this compound was only slightly affected: increased in White Riesling and decreased in Gewürztraminer. On the other hand, bunch rot induced a major increase in the FD factors of 2,3-butanedione with three and four dilution steps in White Riesling and Red Riesling, respectively, while it remained unaffected in Gewürztraminer.

Regarding bunch rot effects on the substance class of aldehydes, methional was increased by three dilution factors in Gewürztraminer. Meanwhile, in White Riesling only a slight increase was observed and Red Riesling was unaffected. In addition, the FD factors of vanillin were increased by four dilution steps in White Riesling and Gewürztraminer, but this substance was not affected in Red Riesling.

Finally, three unknown compounds suffered relevant changes due to bunch rot. First and foremost, the FD factor of the cheesy odorant (RI 1433 on DB-FFAP and 1097 on DB-5) was decreased from FD 128 to 8 in Red Riesling while it was highly increased in Gewürztraminer where it was not detected in HW but perceived with an FD factor of 128 in BW. Second, FD factors of the onion-like smelling substance (RI 1693 on DB-FFAP and 953 on DB-5) were decreased by three and five dilution steps in White Riesling and Red Riesling, respectively. Third, the FD factor of the woody, coconut-like and minty smelling compound (RI 2245 on DB-FFAP and 1311 on DB-5) was decreased by seven dilution steps in White Riesling and increased by four in Red Riesling.

### Effects of powdery mildew infection

The changes on the intensities of the acidic compounds induced by powdery mildew were, in general, subtle. More specifically, the FD factors of acetic, propanoic, isobutanoic, butanoic, hexanoic, and undecanoic acid in PW were equal to those in the corresponding healthy sample. Furthermore, FD factors of 3-methylbutanoic and octanoic acid were subtly decreased while that of phenylacetic acid was slightly increased. Only decanoic acid was exceptional insofar, as the FD factor of this substance showed a relevant decrease in PW by three dilution steps.

Powdery mildew further affected the FD factors of the esters: ethyl isobutanoate, ethyl 2-methylbutanoate, and 3-methylbutyl acetate were decreased to a relevant extent with their FD factors being three dilution steps lower in PW. On the other hand, ethyl isovalerate was not perceived in the healthy sample while it was detected with an FD factor of 16 in PW. Ethyl hexanoate, ethyl octanoate, diethyl succinate, ethyl butanoate, geranyl acetate, phenylacetate and 2-phenylethyl acetate suffered no important modifications with regard to their FD factors.

In the group of the lactones, only moderate changes were observed. The FD factors of γ-nonalactone, γ-undecalactone and sotolone were slightly increased while the intensity of (*Z*)-6-dodeceno-γ-lactone was slightly decreased, and γ-decalactone remained unaffected.

Infection with powdery mildew caused no relevant modifications in the FD factors of the following alcoholic compounds: isobutanol, isoamyl alcohol, (*Z*)-3-hexen-1-ol, 1-hexanol, methionol, linalool, benzyl alcohol, 2-phenylethanol, 4-ethylguaiacol, furaneol and homofuraneol. On the other hand, relevant effects were observed on some phenolic compounds. Thereby, the FD factor of m-cresol was increased by three dilution steps, while 4-ethylphenol and 4-vinylguaiacol were decreased by four and three dilutions steps, respectively.

Regarding the effects on the ketones 2,3-butanedione and 4-mercapto-4-methyl-2-pentanone, no relevant changes in their FD factors were observed.

Lastly, in relation to the influence of the fungus on aldehydes, methional remained unaffected while the FD factor of vanillin was decreased by three dilution steps.

### Sensory evaluation

**Figure 1:**
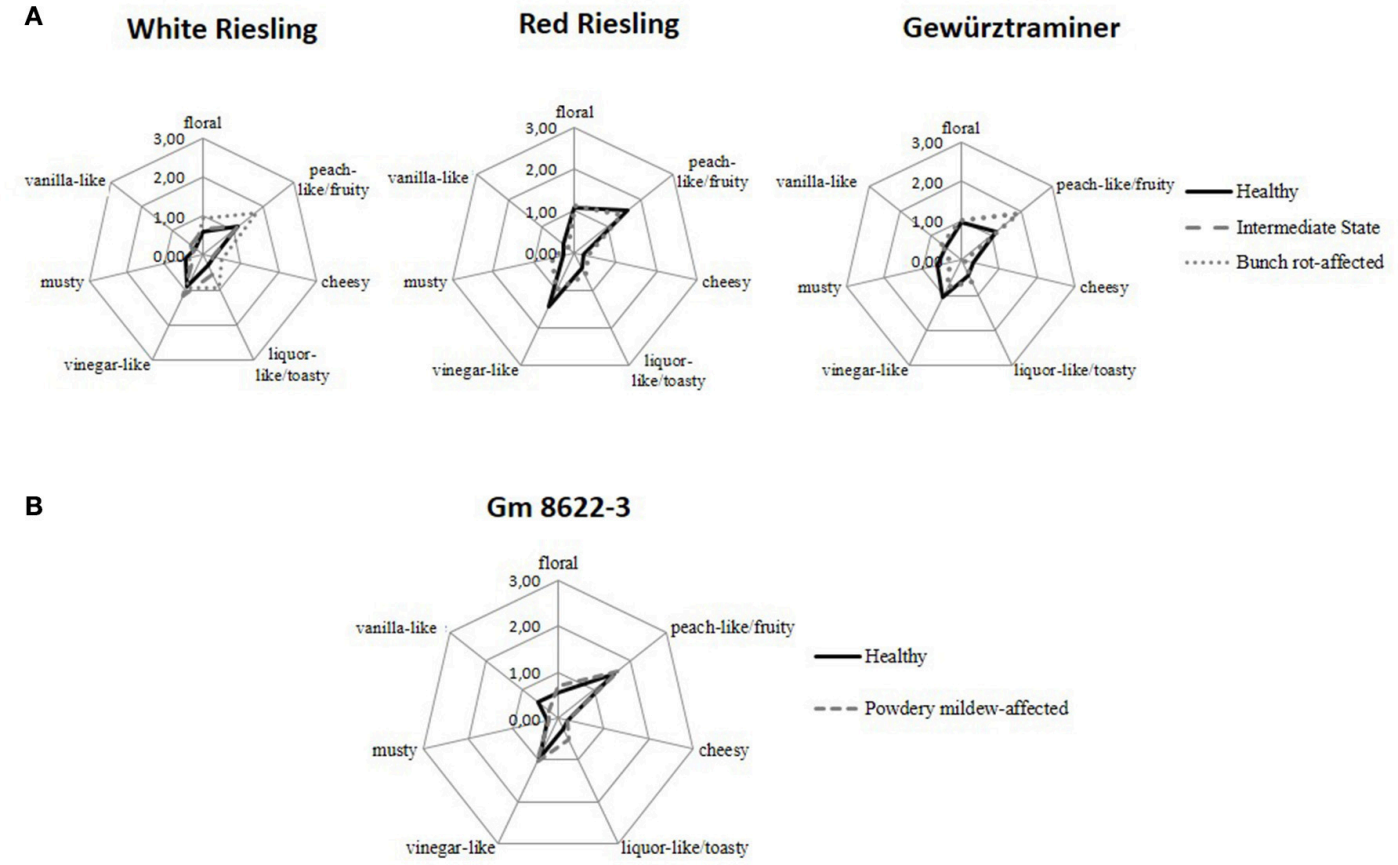
**Results of aroma profile analysis**. Displayed are the means of 10 participants (1 male and 9 females, 25–35 years old). **(A)** Comparison between healthy and bunch rot-affected samples of White Riesling, Red Riesling and Gewürztraminer varieties. **(B)** Comparison between healthy and powdery mildew-affected sample of the hybrid Gm 8622-3.

All wines were evaluated by an expert sensory panel by means of comparative aroma profile analyses (Figures 1A,B). In the course of this evaluation, White Riesling and Gewürztraminer bunch rot-infected samples were perceived as being more *peach-like/fruity*: the intensity rating of this attribute changed from 1.2 for HW to 1.8 and 1.9 for the fungi affected samples of White Riesling and Gewürztraminer, respectively. In the case of Red Riesling, however, this attribute was rated in both samples with similar intensities of 1.5, on average. In addition, the fungus-affected White Riesling sample was perceived as slightly more *floral* with a rating of 0.6 in HW and 1.0 in BW. Meanwhile, no clear differences were observed in Red Riesling and Gewürztraminer varieties, where the intensity of this attribute was rated with 1.0, on average. In addition, the *liquor-like/toasty* note was also intensified in BW: in White Riesling its intensity was increased from 0.3 to 1.0 as a result of the infection whereas just a slight increase from 0.4 to 0.6 of this aroma note was recorded for Red Riesling and Gewürztraminer. The remaining attributes were rated similarly in HW and BW (rating on average: *vinegar-like* 1.2, *cheesy* 0.3, *musty* and *vanilla-like* 0.4, respectively).

Sensory rating of the effects of powdery mildew on the aroma character of the wine showed that the *vanilla-like* note was slightly lower in the fungus-affected sample (0.6 in HW and 0.3 in PW). The remaining attributes were rated similarly in both samples with the following mean values: the *floral* note with 0.6, *peach-like/fruity* with 1.6, *cheesy* with 0.2, *liquor-like/toasty* with 0.4, *vinegar-like* with 1.0 and *musty* with 0.2, respectively.

**Figure 2:**
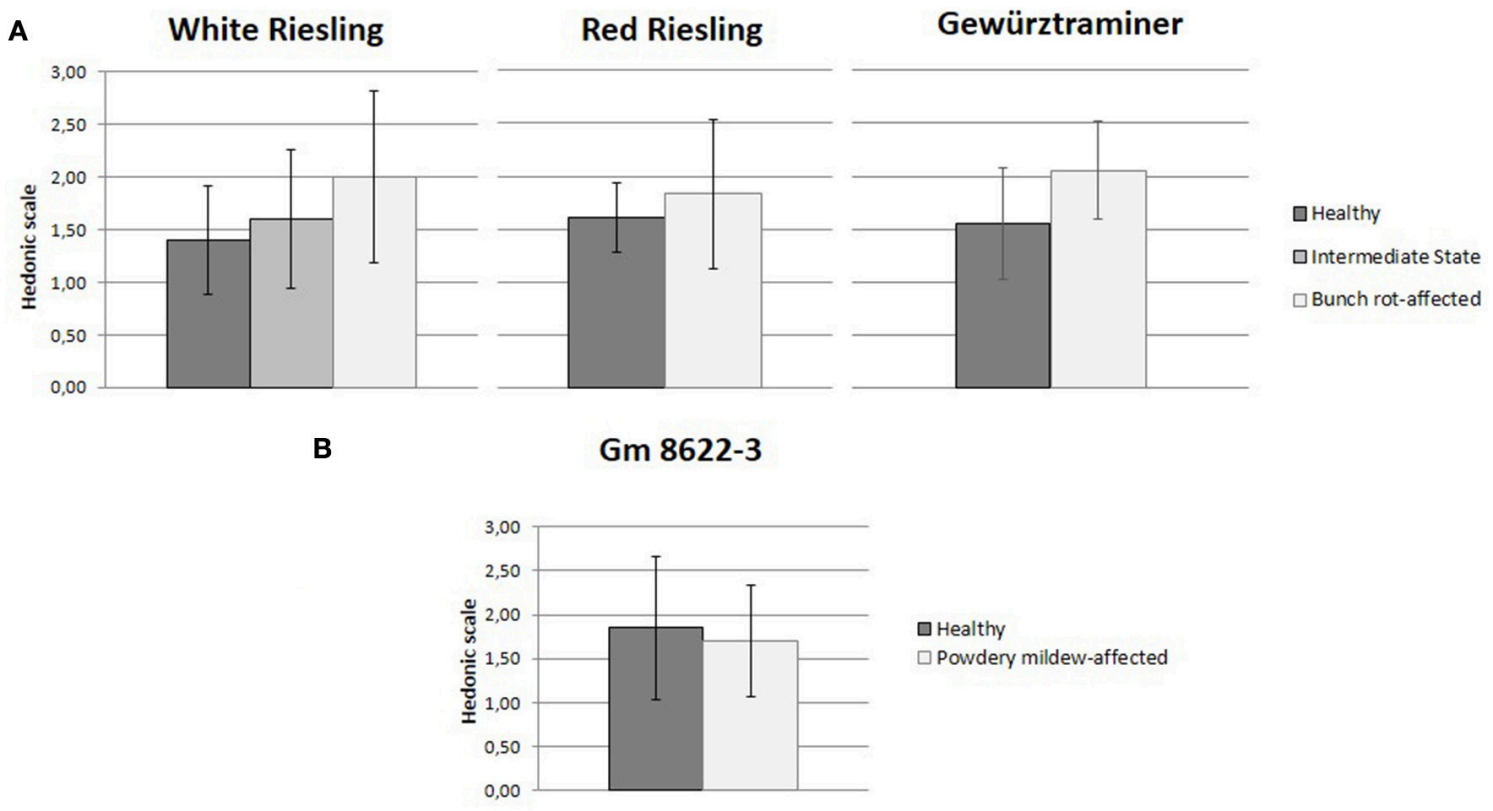
**Results of the hedonic evaluation**. Displayed are the means of 10 participants (1 male and 9 females, 25–35 years old). **(A)** Comparison between healthy and bunch rot-affected samples of White Riesling, Red Riesling and Gewürztraminer varieties. **(B)** Comparison between healthy and powdery mildew-affected sample of the hybrid Gm 8622-3.

With regards to the hedonic evaluation, the *Botrytis* bunch rot-affected samples were rated as being more positive than its corresponding healthy control in all three varieties studied. Thereby, the overall aroma of the HW samples was rated with 1.5, on average, as being moderately pleasant, and of the BW samples as being pleasant with 2.0. The opposite trend was observed in case of powdery mildew, where the affected sample was rated as being more unpleasant than its healthy control: the hedonic impression of HW was rated with 1.9 and PW with 1.7. In the hedonic rating, the variance amongst individuals was pronounced (Figures 2A,B). Overall, however, it was found that due to the high inter-individual variation, the observed sensory differences between samples were statistically not significant.

## Discussion

During fermentation, yeasts are responsible for the transformation of sugar to ethanol and carbon dioxide, and additionally generate a multitude of by-products in the course of these processes. Consequently, fermentation is the main source of wine aroma compounds involving a huge variety of enzymatic steps and pathways (Schreier and Jennings, 1979; Etievant and Maarse, 1991; Rodrıguez-Bencomo et al., 2002). This general consideration is reflected when comparing AEDA results obtained for wine in this investigation with those data obtained for the corresponding must samples in our previous study (Lopez Pinar et al., 2016): overall, the FD factors of a series of compounds were much higher in wine. Thereby, the most potent compounds in the must samples with FD factor 512 were methional and vanillin. Meanwhile, the most intense substances in the wines, isoamyl alcohol and 2-phenylethanol, reached much higher FD factors of 8192.

AEDA results revealed important effects of both fungi infections on FD factors of diverse substances. First and foremost, bunch rot infection caused a general increase in the *fruity*-smelling lactones γ-decalactone, γ-nonalactone, γ-undecalactone, (*Z*)-6-dodeceno-γ-lactone, and the *curry-like* smelling lactone sotolone. This augmentative effect had already been observed in our previous study on the corresponding musts (Lopez Pinar et al., 2016). This observation is supported by findings of other groups showing that *fruity* lactones are amongst the most characteristic aroma components of Tokaji Aszú noble rot wines (Schreier et al., 1976; Miklosy and Kerenyi, 2004; Miklosy et al., 2004). This increase in lactone content might further explain the higher intensity rating of the *peach-like/fruity* attribute in BW in the course of the comparative aroma profile analyses between healthy and infected samples.

In addition, bunch rot development led to highly elevated FD factors in case of vanillin in wine, likewise corresponding to the effect observed in the must samples. However; this increase did not reveal a direct correlation with the rating of the *vanilla-like* note in the sensory tests, since this attribute was rated similarly in healthy and affected samples with an intensity of 0.4. On the other hand, rating of this attribute was fairly variable between panelists. One reason for this variation might be that the *vanilla-like* note might be additionally impacted by other compounds such as *sweet-fruity* impressions from lactones or esters in the course of multi-sensory integration of this complex aroma percept (Lee and Noble, 2003; Prescott, 2012).

Bunch rot further induced a moderate increase in the FD factors of isobutanol, isoamyl alcohol, furaneol, and homofuraneol. These results are also in line with the effects observed in the study on the corresponding must samples. The higher content in these compounds might explain the characteristic *liquor-like/toasty* note perceived in BW. These findings are further supported by a study of Sarrazin et al. (2007) where furaneol and homofuraneol contributed to the aroma of Sauternes botrytized wines.

Likewise, the panelists recorded a more intense *floral* note in White Riesling BW, whereas this intensification was only subtle in the case of Red Riesling and Gewürztraminer. This effect might relate to the sum of diverse minor increases in the FD factors of a series of substances: phenylacetic acid, 2-phenylethanol, phenyl acetate and 2-phenylethyl acetate. Moreover, the FD factors of 2-phenylethanol and phenylacetic acid were also higher in the must samples affected by bunch rot.

The observed differences between bunch rot-affected and healthy samples could be partially related to the higher sugar content reported in the infected grapes (Lopez Pinar et al., 2016). It can be assumed that an increase in sugar content in grape must might imply a more intense fermentation and therefore, a higher content of esters and lactones in wine (Cãmara et al., 2006; García-Martín et al., 2010). In addition, an increase in sugar may be linked to a rise in sugar-derived compounds, such as furaneol and homofuraneol (Sanz et al., 1995; Cãmara et al., 2006). On the other hand, *Botrytis cinerea* enhances the production of laccase, an enzyme exerting an oxidative activity (Slomczynski et al., 1995; Pezet, 1998). Thereby, an increase of isoamyl alcohol and isobutanol content has been attributed to an enhancement of the oxidative deamination of their correspondent free amino acid precursors, leucine and valine, respectively (Cãmara et al., 2006).

Powdery mildew, on the other hand, led to a decrease of the *vanilla-like* note in the affected sample corresponding to a decrease in the FD factor of vanillin in PW by three dilution steps.

The remaining sensory attributes (*floral, peach-like/fruity, cheesy, liquor-like/toasty, vinegar-like*, and *musty*) were rated very similarly in PW and in its corresponding healthy sample. In agreement with this result, the FD factors of the acidic compounds, lactones and esters remained, in general, either unaffected or were only slightly modified.

Regarding the hedonic evaluation of healthy and bunch rot-affected samples, it turned out that the aroma profiles of BW samples were rated as being more pleasant than those of the HW samples in case of all three grape varieties. This effect might be linked to the enhancement of positive aroma attributes (*floral, peach-like/fruity* and *liquor-like/toasty*) caused by bunch rot. On the contrary, the sample affected by powdery mildew was consistently evaluated as being more negative with regard to hedonics. This negative rating was, however, not related to any specific off-note but was rather due to a lack of positive aromatic notes; in fact, the wine was described as being rather flat.

As already mentioned, none of the differences observed in the sensory evaluation of the wines was statistically significant and this was mainly linked to the high variance among participants. Indeed, it is well known that odor perception can be prone to pronounced inter-individual differences (Lorber et al., 2016). These variations may be, amongst others, related to inter-individual differences in the olfactory receptor repertoire or differences in the metabolism of odorants within the nasal cavity (Zhang et al., 2005; Keller et al., 2007; Lorber et al., 2016)

The previously reported *earthy* off-odors caused by *Botrytis* bunch rot and powdery mildew infections (Darriet et al., 2002; Stummer et al., 2005; La Guerche et al., 2006; Steel et al., 2013) were not observed in the course of this study. This deviation might be due to differences in the climatic conditions, an important aspect that would require more detailed elucidation in future studies. In the course of the must analyses, however, six compounds reminiscent of *wet earth* and *fungi* had been detected by GC-O (Lopez Pinar et al., 2016). These had been identified as geosmin, 2-methylisoborneol, 1-octen-3-one, 1-octen-3-ol, 2,3,5-trimethylpyrazine and 3-sec-butyl-2-methoxypyrazine. In the frame of AEDA, these compounds had been perceived only with low FD factors in the musts. Only the FD factor of 2-methylisoborneol reached a relatively high maximum value of 128. As this substance was not detected with relevant FD in the present investigation in wine, it is likely that it has been degraded during fermentation. This assumption is supported by a study of La Guerche et al. (2006) describing the instability of 2-methylisoborneol during fermentation of must, and by a previous study performed by our group showing that this substance is degraded into two odorless dehydration products in wine, namely 2-methylenebornane and 2-methyl-2-bornene (Lopez Pinar et al., 2017).

As the aroma attribute *musty* was an important term in our previous investigation on the aroma profiles of the must samples, it was also included in the present study. Nevertheless, this note was only perceived with a very weak intensity (on average with an intensity of 0.4) which is in line with the lack of detection of any musty smelling substances in the course of AEDA.

## Conclusions

Combinatorial application of comparative AEDA and sensory analysis revealed distinct aromatic differences caused by both fungal infections. These were not associated with any specific key compound, but rather quantitative changes of a series of aroma active substances in the respective wines. In addition, most differences observed for the wine aroma were in line with the changes in odorant composition that had been previously recorded for the corresponding grape musts.

Thereby, bunch rot predominantly induced an increase in the aroma intensities of *peach-like/fruity, floral* and *liquor-like/toasty* notes while powdery mildew caused a decrease in the *vanilla-like* attribute. These effects could be generally linked with changes in substance composition as observed via AEDA. However, bunch rot led to inconsistent effects on the aroma composition in the three investigated varieties demonstrating that both the type of fungal infection as well as the type of grape variety influence the final product to a major extent. Undoubtedly, further research is needed to investigate the cause for the detected differences.

This study contributes to our knowledge on the aroma effects of these two economically relevant fungal infections, and builds the foundation for future more targeted studies, potentially involving quantitative determinations of representative marker substances in relation to these fungal infections and their development under diverse climatic conditions.

## Author contributions

Each author has participated sufficiently in the work, intellectually or practically, to take public responsibility for the content of this article, including the conception, design, and conduct of the experiment and data analysis and interpretation. AL, DR, and ER were responsible for sampling. AL, DR, and ER carried out the practical work. AL, DR, and ER were responsible for data analysis. AB, AL, DR, and ER conceived the study, and AB, AL, DR, and ER participated in the design of the study. All authors contributed to the manuscript and approved the final version.

## Funding

The project was funded by the Bavarian Research Foundation in the project 152-12.

### Conflict of interest statement

The authors declare that the research was conducted in the absence of any commercial or financial relationships that could be construed as a potential conflict of interest.

## Footnotes

^1^EUR-lex (Acces to European law) Communication from the Commission to the Council, the European Parliament and the Economic and Social Committee - Towards a Thematic Strategy on the Sustainable Use of Pesticides (2002), http://eur-lex.europa.eu/legal-content/EN/TXT/?qid=1469694189660&uri=CELEX:52006SC0894 Accessed 28.07.2016.
